# Identification of Novel Conjugative Plasmids with Multiple Copies of *fosB* that Confer High-Level Fosfomycin Resistance to Vancomycin-Resistant Enterococci

**DOI:** 10.3389/fmicb.2017.01541

**Published:** 2017-08-15

**Authors:** Lingyan Sun, Ping Zhang, Tingting Qu, Yan Chen, Xiaoting Hua, Keren Shi, Yunsong Yu

**Affiliations:** ^1^Department of Infectious Diseases, Sir Run Run Shaw Hospital, College of Medicine, Zhejiang University Hangzhou, China; ^2^Key Laboratory of Microbial Technology and Bioinformatics of Zhejiang Province Hangzhou, China; ^3^State Key Laboratory for Diagnosis and Treatment of Infectious Disease, First Affiliated Hospital, College of Medicine, Zhejiang University Hangzhou, China

**Keywords:** *fosB*, fosfomycin resistance, vancomycin resistant enterococci, single-molecule real-time sequencing, copy number

## Abstract

To further characterize the *fosB*-carrying plasmids of 19 vancomycin-resistant enterococci, the complete sequences of the *fosB*- and *vanA*-containing plasmids of *Enterococcus faecium* (pEMA120) and *E. avium* (pEA19081) were obtained by single-molecule, real-time sequencing. We found that these two plasmids are essentially identical (99.99% nucleotide sequence identity), which proved the possibility of interspecies transmission. Comparative analysis of the plasmids revealed that the backbone of pEMA120 is 99% similar to a conjugative *fosB*-negative *E. faecium* plasmid, pZB18. There is a *traE* disrupted in the transfer region of pEMA120, in comparison to pZB18 with an intact *traE*. The difference of their transfer frequencies between pEMA120 and pZB18 suggests this interruption of *traE* might affect conjugative transfer. Two copies of the *fosB* gene linked to a *tnpA* gene, forming an IS*L3*-like transposon, were found at separate locations within pEMA120, which had not been reported previously. These two *fosB*-carrying transposons were confirmed to form circular intermediates by inverse PCR. The hybridization of plasmid DNA digested by *Bsa*I, having restriction site within the *fosB* sequence, demonstrated that the presence of multiple copies of *fosB* per plasmid is common. The total copy number of the *fosB* gene as revealed by qRT-PCR did not correlate with fosfomycin MICs or growth rates at sub-MICs of fosfomycin in different transconjugants. From susceptibility tests, the *fosB* gene, regardless of the copy number, conferred high fosfomycin MICs that ranged from 16384 to 65536 μg/ml. This first complete nucleotide sequence of a plasmid carrying two copies of *fosB* in VRE suggests that the *fosB* gene can transfer to multiple loci of plasmids by the IS*L3* family transposase TnpA, possibly in the form of circular intermediates, leading to the dissemination of high fosfomycin resistance in VRE.

## Introduction

Nowadays, the colonization and infection of vancomycin-resistant enterococci (VRE) is a global public health concern due to the increasing dissemination of these bacteria and limited clinical treatment options (Ranotkar et al., 2014). Fosfomycin, a drug discovered in 1969 that is primarily used to treat uncomplicated urinary tract infections (Keating, 2013), has been shown to be effective against VRE (Superti et al., 2009; Descourouez et al., 2013; Tang et al., 2013). Therefore, fosfomycin has gained attention as a potential therapeutic option for treating VRE infections (Popovic et al., 2010). However, resistance to fosfomycin emerges rapidly, likely because of the high frequency at which fosfomycin-resistant mutants arise (Greenwood, 1990; Hamilton-Miller, 1992). Therefore, additional studies on fosfomycin resistance mechanisms are required to better guide clinical practices. Until now, the fosfomycin resistance enzyme FosB, which catalyzes the Mg(II)-dependent addition of L-cysteine, or bacillithiol, to the epoxide ring of fosfomycin (Thompson et al., 2014), was the only known plasmid-borne fosfomycin-resistance determinant in *Enterococcus* spp. (Xu et al., 2013; Qu et al., 2014). The *fosB* gene was reported to be located on a transferable circular intermediate (Xu et al., 2013). Additionally, the *fosB* gene has also been reported to be present in plasmids of *Staphylococcus* spp. (99% nucleotide similarity) (Zilhao and Courvalin, 1990; Fu et al., 2016) and in the chromosome of *Bacillus subtilis* (63% nucleotide similarity) (Cao et al., 2001). In our previous study, we characterized 18 VRE isolates that were resistant to fosfomycin (Qu et al., 2014). The plasmid-encoded *fosB* gene inserted into the *vanA* type Tn*1546*-like transposon, likely accounts substantially for the high fosfomycin resistance rate observed in VRE. This paper reports on the detailed plasmid environment of *fosB* gene by third-generation sequencing and the formation of circular *fosB* intermediates. We also discuss the contribution of *fosB* copy number on the fosfomycin MIC and bacterial growth rate at a sub-MIC level of fosfomycin in the assayed VRE strains.

## Materials and Methods

### Bacterial Isolates

*Enterococcus faecium* A120 and 17 additional *fosB*-carrying, vancomycin resistant *E. faecium* isolates were characterized in our previous paper (Qu et al., 2014). *E. avium* 19081 is a clinical isolate that was collected during the same time period from fecal specimens carrying *fosB* and *vanA*. Using these isolates as donor strains, fosfomycin resistant transconjugants were obtained by filter mating experiments using *E. faecium* BM4105RF as the recipient strain (Qu et al., 2014).

### Antimicrobial Susceptibility Testing

The MICs of fosfomycin against 19 VRE isolates and their respective *E. faecium* BM4105RF transconjugants were determined by the agar dilution method. *E. faecalis* ATCC 29212 was used as the control. The results were interpreted according to the Clinical and Laboratory Standards Institute (CLSI) 2015 guidelines (CLSI, 2015).

### Conjugation Experiment

Broth mating and filter mating of *E. faecium* A120 were performed to get the transfer frequency of plasmid pEMA120 using *E. faecium* BM4105RF as the recipient strain with a donor/recipient ratio of 1:10 as previously described (Zheng et al., 2007).

### Bacterial Growth at Sub-MIC Levels

Single colonies were grown at 37°C with shaking at 200 r.p.m. in 2 ml of brain heart infusion (BHI) broth. Independent overnight cultures were diluted (1:1000) and 200 μl was transferred to a 96-well plate that was incubated at 37°C. The turbidity was measured at OD_600_ using a BioTEK Synergy plate reader (BioTEK, Winooski, VT, United States) every 5 min for 16 h.

A preliminary experiment using isolate A120 was carried out using a fosfomycin concentration gradient from 1/128 the MIC to the MIC. The growth of the 19 transconjugants that were exposed to 1/4 the MIC of fosfomycin was measured and compared with an antibiotic-free control. Each sample was performed in triplicate.

### Plasmid Isolation, *Bsa*I Restriction Enzyme Digestion and Southern Blot Hybridization

Plasmids were isolated from the 19 transconjugants using a QIAGEN Plasmid Midi Kit (Qiagen, Germany). The manufacturer’s instructions were followed with the inclusion of the lysozyme treatment (10 mg/mL, 60 min at 37°C) prior to the lysis step with buffer P2 [200 mM NaOH, 1% SDS (w/v)].

The restriction enzyme *Bsa*I (New England Biolabs, United States), for which a target site is present within the *fosB* gene, was used to digest plasmid DNA. The hybridization probe was designed to bind within the latter half of the *fosB* gene after *Bsa*I digestion (Supplementary Figure S1A) and was synthesized using the primers *fosB*-latter-F (5′-GTG GTA TAT GGT TAG CTT TGA ACG AAG-3′) and *fosB*-latter-R (5′-TGA GGT TTA GCC TCT TTA TAA TAA CTC-3′).

Plasmid DNA was first digested by *Bsa*I at 37°C in a water bath overnight. After the digested DNA was agarose gel electrophoresed for 2 h, the DNA fragments were transferred to a positively charged nylon membrane (Millipore, United States), hybridized with a DIG-labeled *fosB*-latter specific probe, and then was detected with a NBT/BCIP color detection kit (Roche, Germany).

### Quantification of the *fosB* Gene by Real-Time PCR Amplification

The copy number of *fosB* was measured via quantitative real-time PCR (qRT-PCR). The chromosomal housekeeping gene *purK* was chosen as an internal control. The sequences of the *fosB* and *purK* primers used are as follows:

Q-*fosB*-F (5′-CTCAATCTATCTTCTAAACTTCCTG-3′),
Q-*fosB*-R (5′-CGATTTTGAAGATTGGTATAACTGG-3′),
Q-*purK*-F (5′-GATATCCAAGATGCGATTGAC-3′), and
Q-*purK*-R (5′-CTTCTAAAACACAGGTTCCTTCTC-3′).

Quantitative PCR reactions were carried out in a 10 μl reaction that contained 5 μl SYBR^®^ Premix Ex Taq^TM^ PCR kit (Takara Bio, Japan), 2 ng of genomic DNA as template, and 2 pmol of each primer. The Ct values of each sample was measured under appropriate PCR conditions (preheated at 95°C for 5 min; 40 amplification cycles at 95°C for 5 s, 54°C for 30 s and 72°C for 30 s) on an Applied Biosystems ViiA^TM^ 7 Dx instrument (Applied Biosystems, United States). Data were calculated based on the 2^-ΔΔC_t_^ method.

### DNA Sequencing and Analysis

Single-molecule, real-time sequencing (Pacific Biosciences, United States) was carried out to complete the whole genome sequencing of *E. faecium* pEMA120 and *E. avium* pEA19081 plasmids. The plasmid sequences were validated by PCR, annotated using the RAST server (Aziz et al., 2008) and were supplemented by the BLASTP program. Lastly, the circular map of the pEMA120 and pEA19081 plasmids were generated using the CGview server (Grant and Stothard, 2008). A comparison of pEMA120 and three related plasmids was made with Mauve 2.3.1 and modified by Photoshop 7.0.

Inverse PCR was performed using the primers fosBiF (5′-TGTCAGCCCCTAAAATATCTCT-3′, located within the *fosB* gene) and fosBiR (5′-GTTTCAAATGTACCTAAAGAACT-3′, located within the *tnpA* gene) (Xu et al., 2013) with genomic DNA as template. The purified PCR products were cloned into the pMD20-T Vector (Takara Bio, Japan) and were sequenced (Biosune, China).

## Results

### Plasmid Structure

**FIGURE 1:**
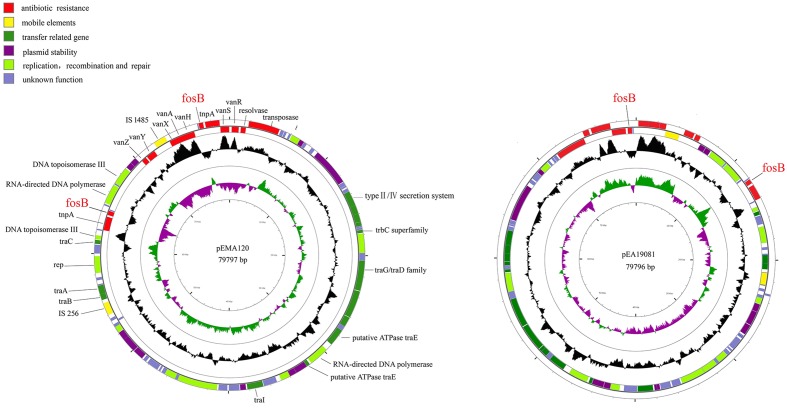
Structure of plasmid pEMA120 and pEA19081. The two outer circles represent ORFs in the plus (outside) and minus (inside) orientations, respectively. Functions are color coded as explained in the key. The two inner circles represent the G + C content plotted against the average G + C content of 33.8% (black circle) and GC skew information (green and purple circles).

To further characterize the previously identified *fosB*-carrying plasmids, the plasmids from two VRE isolates (*E. faecium* A120 and *E. avium* 19081) were completely sequenced. Plasmid pEMA120 is 79,797 bp in size with an average G + C content of 33.8%. It contains 87 open reading frames (ORFs), 47 of which encode proteins with homology to proteins with known functions, which can be classified into several functional modules: antibiotic resistance, mobile elements, plasmid stability, replication recombination and repair, and conjugal transfer (**Figure 1**).

**FIGURE 2:**
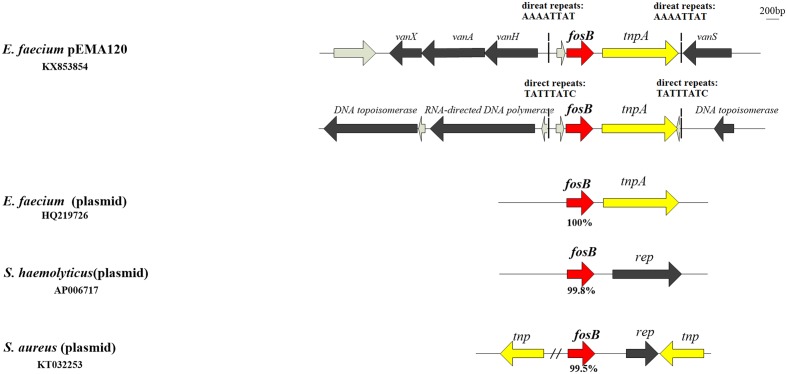
The *fosB* gene and genetic environment of *fosB* in different species or genera. *fosB* genes are indicated by red arrows with nucleotide identity below.

In the antibiotic resistance region, there are two types of resistance determinants: *fosB* mediating fosfomycin resistance and *vanA*-type transposon causing vancomycin resistance. Two copies of *fosB* gene along with *tnpA* insert into different position of pEMA120 leading to diverse direct repeats (**Figure 2**). One *fosB* gene along with *tnpA* gene reversely inserts into the *vanA*-type Tn*1546*-like transposon between the *vanRS* and *vanHAX* intergenic region. Another *fosB* gene accompanied with *tnpA* gene is flanked by genes encoding a DNA topoisomerase and a RNA-directed DNA polymerase, which are required for replication, recombination and repair. No same structure was found in existing database, indicating that this is a novel insertion position of the *fosB* gene.

The surrounding environment of *fosB* on pEMA120 was compared with *fosB* in other species (**Figure 2**) Plasmid-borne *fosB* in *Staphylococcus haemolyticus* and *Staphylococcus aureus* share 99.8 and 99.5% nucleotide identity with *fosB* in the plasmid of *E. faecium*, respectively. As to surrounding environment, there is a *rep* gene downstream *fosB* on *Staphylococcus* spp. plasmid instead of a *tnpA* gene adjacent to *fosB* on *E. faecium* plasmids.

The conjugal transfer region of pEMA120 has some genes that are characteristic of a conjugative plasmid. The genes of ORF60 and ORF61, respectively, show similarity with 43 and 58% identity (at the amino acid level) to *traB* and *traA* of the conjugative plasmid pMG1 (Tanimoto and Ike, 2008). There is a predicted interrupted gene comprised of ORF29 and ORF31 that encodes a putative ATPase TraE, which belongs to the motor protein family of the type IV secretion system (T4SS) in enterococci (Chandran et al., 2009; Clewell et al., 2014). The *traI* gene (ORF38) shows 71% identity at the amino acid level with ORF34 of pHTβ encoding the putative DNA relaxase/nickase, which is needed for the initiation of DNA transfer in conjugative plasmids (Francia et al., 2004).

As regards plasmid replicon typing, ORF64 showed 57% identity at the amino acid level with Rep protein of plasmid pHTβ from *E. faecium* and showed less than 80% identity, at both nucleotide and amino acid level, with any plasmid replicon type according to the classification system of enterococci (Jensen et al., 2010).

**FIGURE 3:**
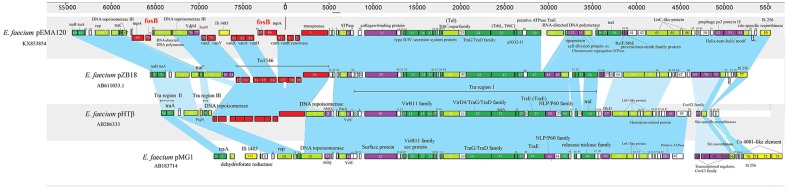
Comparison of *E. faecium* plasmid pEMA120 and three reported *E. faecium* plasmids pZB18, pHTβ, pMG1. Light blue shades indicate shared regions with a high degree of homology. ORFs are portrayed by rectangles and colored according to their putative functions. Light-green rectangles indicate genes associated with replication, recombination and repair. Genes associated with plasmid conjugal transfers are colored in green. Genes involved in plasmid stability are indicated by purple. Red and yellow rectangles indicate antimicrobial resistance genes and mobile elements genes, respectively. White rectangles indicate genes for hypothetical proteins as well as proteins of unknown function.

To better study the evolution of pEMA120, we compared it to three similar *E. faecium* plasmids reported previously (**Figure 3**). Plasmid pMG1 (GenBank accession no. AB183714) is a gentamicin and kanamycin resistance plasmid capable of highly efficient transfer among enterococci and originally isolated from a clinical strain of *E. faecium* in Japan, 1998 (Ike et al., 1998). Plasmid pHTβ(GenBank accession no. AB206333) is a vancomycin resistance pMG1-like plasmid which was obtained from a hospital in the United States, 2003 (Tomita et al., 2003). Plasmid pZB18 (GenBank accession no. AB611033.1) acquired from Beijing, 2004 (Zheng et al., 2007) is also a vancomycin resistance conjugative *E. faecium* plasmid but without conserved *traA* gene or *traB* specific for pMG1-like plasmid. The distinctive feature of pEMA120 is the structure of the *fosB* connected by *tnpA*, which does not exist in other three plasmids. In general, the backbones of the four plasmids have high homology, which is depicted in the figure as the blue shades. In particular, plasmid pZB18 shares 99% identity and 85% query cover with pEMA120. Plasmid pMG1 and pHTβ contain highly efficient conjugative system, especially the similar oriT and transfer-related regions, which have a high degree of homology with the conjugal transfer region of pEMA120.

As shown in **Figure 1**, the *E. avium* plasmid pEA19081 was found to share 99.99% nucleotide sequence identity with the *E. faecium* plasmid pEMA120 after sequence comparison by BLASTN. Thus, the two plasmids are virtually identical, containing the same two copies of *fosB* as well as a Tn*1546* transposon.

### The Effect of Interruption of *traE* on the Conjugative Transfer

As mentioned in above section, a transfer related gene *traE* which might play an important role in conjugal transfer process, was disrupted by a gene encoding RNA-directed DNA polymerase. To investigate whether this interruption could affect the conjugative transfer, we compared the transfer frequency of plasmid pEMA120 with a disrupted *traE* and plasmid pZB18 with an intact *traE*. From Supplementary Table S1, the transfer frequency of pEMA120 was distinctly lower than that of pZB18, using broth mating or filter mating.

### The *fosB*-Carrying Circular Intermediate

**FIGURE 4:**
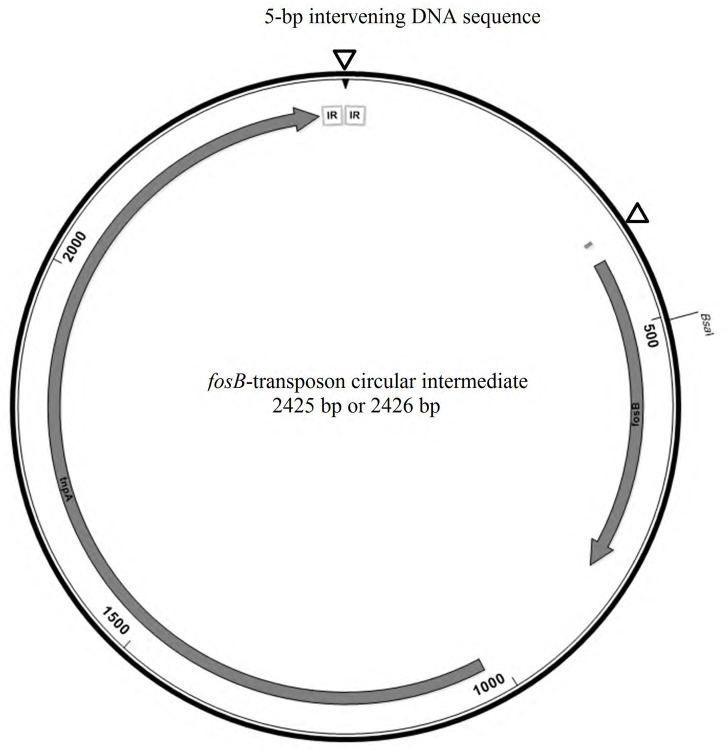
The schematic map of *fosB*-transposon circular intermediate. The polymorphism regions were indicated by triangles. The two IRs sequence was framed in gray.

To determine whether *fosB* gene-containing circular intermediates are present in isolate A120, an inverse PCR was performed using previously reported primers (Xu et al., 2013). As expected, a ∼1 kb band was present after agarose gel electrophoresis, indicating the presence of circular intermediates. However, the sequencing electropherogram had imperfect signals, suggesting that this amplified 0.92-kb PCR product was probably a combination of two or more sequences (Supplementary Figure S2). Thus, TA-based cloning and sequencing was employed to obtain unique sequences. All sequences of PCR products that were ligated into pMD20-T vectors aligned similarly except for two regions. The first region was located 26–31 bp upstream of the *fosB* gene, showing two variants (ATTTG or TAACAT) (Supplementary Figure S2A), matching the corresponding positions of each *fosB*-carrying IS*L3*-like transposon within pEMA120. Another polymorphism region, 5-bp in length, was situated between the two IRs of IS*L3*-like transposon, i.e., the circle-junction region. Three kinds of sequences were observed, ATTTT, TATTT, or TTATC, as shown in Figure S2B. The complete *fosB*-transposon circular intermediate is 2425 or 2426 bp in length, consisting of the IS*L3*-like transposon and a 5-bp intervening DNA sequence (**Figure 4**). In addition, this *fosB*-transposon circular intermediate could also be acquired by inverse PCR in the other 18 VRE isolates. However, the circular intermediates could not be detected by southern hybridization (using the *fosB-latter* specific probe) after gel electrophoresis using the uncut or S1 nuclease digested pEMA120 plasmid (data not shown).

### The *fosB* Copy Number in Other *fosB*-Containing Plasmids

Our previous study suggested each of 19 VRE isolates contained one *fosB*-carrying plasmid by performing S1 nuclease-pulsed-field gel electrophoresis (PFGE) (Qu et al., 2014). The third-generation sequence analysis showed that both pEMA120 and pEA19081 carry two copies of the *fosB* gene. To determine the *fosB* copy number in these plasmids from transconjugants of 19 VRE isolates, a special hybridization protocol after a restriction enzyme digestion was used. The principle is depicted in Supplementary Figure S1 using pEMA120 as an example on basis of its clear plasmid sequence.

**FIGURE 5:**
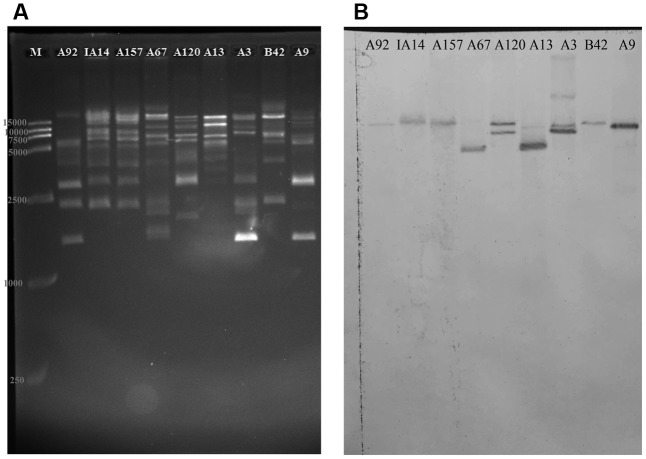
Determination of the *fosB* gene copy number by enzyme *Bsa*I digestion binding hybridization. Shown is **(A)** agarose (1%) gel electrophoresis in 0.5 × Tris-boric acid-EDTA buffer of *Bsa*I-digested plasmids from the transconjugants of A92, IA14, A157, A67, A120, A13, A3, B42, and A9 stained with Gel Red and visualized under UV light, and **(B)** Southern blot hybridization with the *fosB*-latter probe. M is a 15-kb size DNA marker.

As **Figure 5A** shown, the DNA band pattern generated by *Bsa*I digestion showed diverse restriction patterns in the plasmids from the transconjugants of 19 VRE isolates. The hybridization bands of isolate A120 in **Figure 5B** were well in accordance with the simulating graph of pEMA120 in Supplementary Figure S1, which proved the reliability of *Bsa*I-digestion method to explore the *fosB* copy number in a plasmid. The isolates IB3, A165, IA110, 19081, A166, 1001, A96, A155, A158, and IA28, which were analyzed but were not shown here, generated very similar two-band signals (16 and 9.5 kb) with isolate A120. This finding suggests that these isolates bear two *fosB* copies per plasmid. These results were verified by PCR amplification using primers (“between *vanSH*” and “*fosB*-2th”) designed to amplify an approximately 3-kb fragment surrounding each *fosB* (Supplementary Table S2). It was found that all the above isolates contained the two DNA fragments with *fosB*.

The nylon membranes of isolates A67, A13, A9, B42, IA14, A157 and A92 showed a single band, suggesting that there was one *fosB* copy per plasmid.

With regards to isolate A3, four bands were observed on the hybridization membrane. *Bsa*I-digestion electrophoresis and hybridization blotting of A3 showed the possibility that more than one *fosB*-carrying plasmid of similar size existed in at least some isolates. The distribution of the four copies of the *fosB* gene in isolate A3 warrants further investigation using other technologies, such as third-generation sequencing.

### The *fosB* Copy Number Measured by qRT-PCR

To investigate whether *fosB* copy number has any relevance to the fosfomycin MICs and bacterial growth rate at sub-MIC levels, the *fosB* copy number in every transconjugant isolate was determined by qRT-PCR. **Table 1** shows the total *fosB* copy number in every isolate, using the housekeeping gene *purK* as an internal control.

**Table 1:** The *fosB* copy number and fosfomycin MICs without limitation of 19 vancomycin-resistant enterococci isolates.

Strain no.	Copy number of *fosB* gene per plasmid	Total copy number of *fosB* gene [2^-Δ*ct*(*fosB-purK*)^]	Fosfomycin MICs(μg/ml)	
			
			Clinical strains	Transconjugants
IA14	1	4.33 ± 1.77	32768	16384
A157	1	5.23 ± 2.14	32768	16384
A92	1	7.64 ± 0.24	65536	32768
A13	1	1.50 ± 0.23	32768	32768
A67	1	4.06 ± 0.13	32768	65536
B42	1	9.06 ± 3.92	32768	65536
A9	1	188.45 ± 8.39	65536	65536
A120	2	27.44 ± 9.37	65536	32768
19081	2	19.86 ± 3.73	65536	32768
A158	2	25.25 ± 10.97	65536	32768
A96	2	19.52 ± 8.38	65536	32768
IB3	2	17.17 ± 4.36	65536	32768
A165	2	32.70 ± 4.95	65536	32768
A166	2	27.02 ± 4.75	65536	32768
A155	2	24.94 ± 10.65	65536	32768
1001	2	29.50 ± 5.52	131072^a^	32768
IA110	2	34.83 ± 7.14	131072^a^	32768
IA28	2	55.68 ± 8.98	65536	65536
A3	UND^b^	7.59 ± 2.38	65536	65536


*^a^The MIC of 131072 μg/ml was a presumptive value based on the fact that there were some colonies grown on the agar of fosfomycin concentration 65536 μg/ml as the maximum concentration. ^b^Undetermined.*

The total *fosB* copy numbers [2^-Δct(^*^fosB-purK^*^)^] reported in **Table 1** range from 17.17 to 55.68 in the isolates with two-copies of *fosB* per plasmid (transconjugants A165, IA110, 19081, A166, 1001, A96, A155, A158, A120, and IA28). In the A3, A13, A9, A92, B42, IA14, A67, and A157 transconjugants, their total *fosB* copy number was less than 10, except isolate A9 (188.45).

### The Contribution of *fosB* Copy Number to Fosfomycin MICs

The detailed fosfomycin MICs (without an upper limitation) of 19 VRE isolates and their transconjugants is shown in **Table 1**. From the susceptibility tests, the MICs of fosfomycin for these isolates and their transconjugants were extremely high, ranging from 16384 to 131,072 μg/ml (**Table 1**). The MICs of the transconjugants was discussed due to their purer genetic background. As shown in Supplementary Figure S3A, the MIC of fosfomycin did not correlate with the copy number of *fosB* (Pearson’s *R*^2^, 0.1550). For example, although the *fosB* copy number of isolate A67 and B42 were low, their MICs of fosfomycin were higher than most of the other isolates.

### The Effect of *fosB* Copy Number on Bacterial Growth Rate at Sub-MIC Levels

From a preliminary analysis of isolate A120, fosfomycin concentrations of 1/4 or 1/2 the MIC notably affected the bacterial growth, including a prolonging of the lag phase and a decrease in growth rate and max biomass (data not shown). The growth of the 19 transconjugants following exposure to 1/4 MICs of fosfomycin was measured (Supplementary Table S3), but no correlation was observed between the copy number of *fosB* and growth rate, maximum optical density (OD_600_) or lag time (Supplementary Figure S3).

## Discussion

Recently, a series of complete genome sequences of *Enterococcus* have been obtained by high-throughput sequencing (Lam et al., 2012; Boyd et al., 2015; McKenney et al., 2016; Peng et al., 2016). However, the complete sequence of the *fosB*-carrying *Enterococcus* plasmid has not been reported. In this study, the complete sequence of a novel *E. faecium* plasmid containing two copies of *fosB* was obtained by third-generation DNA sequencing technology, with long read lengths obtained. The coexistence of a *vanA* transposon and a *fosB* gene in the same plasmid, which was primarily determined by S1-PFGE in our previous study (Qu et al., 2014), was verified by third-generation sequencing in this report. Furthermore, the fact that *E. avium* 19081 contained a similar *fosB*- and *vanA*-carrying plasmid proved a possibility of interspecies transmission, which could accelerate the dissemination of fosfomycin and vancomycin resistance. The comparison of pEMA120 and three related *E. faecium* plasmids revealed that their plasmid backbones are relatively conserved. In particular, the *fosB*-negative *E. faecium* plasmid pZB18 has a 99% sequence identity with the backbone of pEMA120. It suggested that the two *fosB* genes along with *tnpA* and some other sequences, such as IS*1485* and a gene encoding a retron-type RNA-directed DNA polymerase, were incorporated into plasmid pZB18 to generate pEMA120. Plasmid pZB18 may have evolved from earlier conjugative plasmids, such as pMG1 (Japan, 1998) and pHTβ (United States, 2003) reflecting a stepwise evolutionary process through several recombination events.

Conjugative transfer is an important means of horizontal spread of antibiotic resistance and an important driver in evolution (Frost et al., 2005; Thomas and Nielsen, 2005; Goessweiner-Mohr et al., 2014). The plasmid pEMA120 was tested to be a conjugative plasmid that can transfer from *E. faecium* A120 to the recipient strain *E. faecium* BM4105RF, using filter mating at a rate of 3.4 × 10^-4^ /donor CFU. The tra region of plasmid pEMA120, of highly homology with that of pZB18, has *traB*, *traA*, *traC*, *traI*, *traG*/*D*, *traE* and other transfer related genes. It is noteworthy that the gene encoding TraE of plasmid pEMA120 was disrupted, compared with that of plasmid pZB18. The plasmid transfer rate of pZB18 with an intact *traE* was 7.0 × 10^-1^ (filter mating) as provided by Zheng et al. (2007), obviously higher than that of plasmid pEMA120. It suggests that the interruption of *traE* might affect conjugative transfer and would limit the dissemination of this *fosB*- and *vanA*-carrying plasmid to some extent compared to plasmid pZB18. However, it cannot be ignored that the transfer rate of 3.4 × 10^-4^ of pEMA120 still confer high capability of *fosB*- and *vanA* co-transmission.

The *fosB* gene in the *E. faecium* plasmid has a close phylogenetic relationship with that of *Staphylococcus*, with more than a 99% shared sequence identity. However, the sequence surrounding the *fosB* gene is diverse among different genera. Instead of the *rep* genes that are adjacent to *fosB* in staphylococcal plasmids (Takeuchi et al., 2005; Fu et al., 2016), there is a *tnpA* gene (an IS*L3*-like transposase-encoding gene) downstream of *fosB* that forms an IS*L3*-like transposon in enterococcus plasmids, as previously reported (Chen et al., 2014; Qu et al., 2014). This IS*L3*-like transposon is a 2,516 or 2517-bp element that is flanked by two perfect 25-bp inverse repeats (IRs), which generates two AT-rich 8-bp target duplications upon insertion. IS*L3*-family elements have been reported to be involved in gene inactivation or transcriptional activation of silent genes in *Pseudomonas* strains (Kallastu et al., 1998; Christie-Oleza et al., 2008). Recently, Sivertsen et al. (2016) demonstrated that an IS*L3*-like element insertion upstream of *vanHAX* mediated a silenced VanA phenotype in enterococci. However, in our study, a similar insertion of the *fosB-*carrying IS*L3*-like transposon between *vanRS* and *vanHAX* did not cause the transcriptional inactivation of *vanA*.

In addition to the *fosB* gene within plasmids, the formation of *fosB-*carrying circular intermediates that were 2425 or 2426 bp in length was confirmed by inverse PCR in our isolates, as was reported by Xu et al. (2013). Interestingly, two types of circular intermediates with slight sequence differences (26–31 bp) upstream of the *fosB* gene were observed in the genome of isolate A120, corresponding to the two *fosB* carrying transposons within plasmid pEMA120. This suggested the two *fosB*-carrying transposons are not copies but likely result from two separate insertion events. In addition, the junction region of the loop exhibited sequence diversity in a 5-bp DNA sequence between the two IRs of the IS*L3*-like transposon. The 5-bp intervening DNA sequence was likely derived from the IS flanking regions (Christie-Oleza et al., 2009).

The third generation sequencing has been shown to have advantages in finding multi-copy genes (Guo et al., 2016) because of its genome-wide sequencing scale and high accuracy (McCarthy, 2010). However, the cost of single-molecule, real-time sequencing is high at present. Restriction enzyme digestion and Southern blotting can be a good supplementary tool to explore the copy number of a certain gene in a collection of strains. Here, the hybridization results revealed that the presence of multiple copies of *fosB* per plasmid was a frequent occurrence in VRE isolates (12 of 19). But this method used in our study had a limitation — if the digested fragments of different *fosB* copies were in similar sizes, it would be difficult to separate them clearly by agarose gel electrophoresis and distinguish them by Southern blotting. Therefore we also performed quantitative PCR to determine the total copy number of *fosB* in an isolate. It revealed that the total copy number of *fosB* in isolates carrying two copies of *fosB* per plasmid was more than double that of isolates carrying a single copy of *fosB* per plasmid, possibly due to the higher frequency of *fosB*-carrying circle formation in the former, as was reported previously (Kallastu et al., 1998). The variable total *fosB* copy number in different isolates measured by qRT-PCR might be the result of the variety of three factors: *fosB* copy number per plasmid, plasmid copy number, and probably the amount of *fosB*-carrying circular intermediates. This complicated existence of *fosB* could explain why isolate A9 with only one copy of *fosB* per plasmid has a huge total *fosB* copy number while A3 is the opposite. Thus, multiple research tools, such as whole-genome sequencing, Southern blotting and quantitative PCR, should be conducted to confirm the distribution and copies of *fosB* gene in enterococci.

The copy number of some resistance genes has been reported to affect the MICs of the corresponding antibiotics (Bertini et al., 2007). In our study, however, no correlation between the *fosB* copy number and the fosfomycin MICs was observed. Of note, the fosfomycin MICs observed in this study ranged from 64 to 512 times of 256 μg/ml (the breakpoint of MIC of fosfomycin resistant *Enterococcus*), far exceeding the normal clinical theoretical dose as well as the maximum serum drug concentration (*C*_max_) used for intravenous fosfomycin (606 mg/l) (Roussos et al., 2009). In this case, the influence of the *fosB* copy number on fosfomycin MICs may have minimal practical significance but may be a theoretical reference. However, it is certain that the plasmid-encoded *fosB* gene, even a single copy, can confer high levels of fosfomycin resistance in enterococci. Subinhibitory concentrations of fosfomycin tended to affect the growth of the bacteria by increasing the lag period and decreasing the growth rate and maximum biomass in our study. However, the copy number of *fosB* had little correlation with the variance of bacterial growth rate at sub-MIC levels of fosfomycin in our study. That is to say, the increase in *fosB* copy number did not necessarily help bacterial growth at sub-MIC levels of fosfomycin.

## Conclusion

A novel plasmid with two copies of the *fosB* gene in VRE was first reported in this study. The transfer region and comparative analysis of closely related plasmids suggested that pEMA120 is a pZB18-like conjugative plasmid that has undergone a series of evolutionary changes. The identification of a similar plasmid in *E. avium* proved the possibility of inter-species dissemination of the fosfomycin resistance. The multiple copies of the *fosB* gene in plasmids is common in our VRE isolates. However, plasmid-borne *fosB*, whether single- or multi-copy, can confer a high level fosfomycin resistance. Nevertheless, the *fosB* IS*L3*-like transposon, which can translocate to multiple loci in the plasmid, possibly in the form of circular intermediates, may accelerate the dissemination of fosfomycin resistance in VRE.

## Author Contributions

Conceived and designed the experiments: YY, YC, TQ, and LS; Performed the experiments: LS, PZ, and KS; Analyzed the data: XH and LS; Wrote the manuscript: YY, LS, TQ, YC, and PZ; All authors read and approved the final manuscript.

## Conflict of Interest Statement

The authors declare that the research was conducted in the absence of any commercial or financial relationships that could be construed as a potential conflict of interest.
