# Supplementary material for: Identification of Novel Conjugative Plasmids with Multiple Copies of fosB that Confer High-Level Fosfomycin Resistance to Vancomycin-Resistant Enterococci
Source: Front Microbiol. 2017 Aug 15;8:1541. doi: 10.3389/fmicb.2017.01541 (PMC5559704; doi:10.3389/fmicb.2017.01541)
Supplement: Supplementary file 1 [file Table_1.pdf]

TABLE S1 Conjugation capacity of plasmid pEMA120 with an disrupted *traE* and plasmid pZB18 with an intact *traE*

donors	Transfer frequency of vancomycin resistance (per donor cell)	
	Broth mating (4 h)	Filter mating (16 h)
E. faecium A120	8.3×10^{-9}	3.4×10^{-4}
E. faecium ZB18	[a]3.1×10^{-4}	[a]7.0×10^{-1}

[a] The data of *E. faecium* ZB18 are from (Zheng et al., 2007).
