# Supplementary material for: Identification of Novel Conjugative Plasmids with Multiple Copies of fosB that Confer High-Level Fosfomycin Resistance to Vancomycin-Resistant Enterococci
Source: Front Microbiol. 2017 Aug 15;8:1541. doi: 10.3389/fmicb.2017.01541 (PMC5559704; doi:10.3389/fmicb.2017.01541)
Supplement: Supplementary file 2 [file Table_2.pdf]

TABLE S2 | Primers designed in this study

Primer	DNA sequence (5 ´ to 3 ´)	Length (bp)	Target
fosB-latter-F	GTGGTATATGGTTAGCTTTGAACGAAG	274	*Bsa*I-probe
fosB-latter-R	TGAGGTTTAGCCTCTTTATAATAACTC		
Between *vanSH*-F	GCTCTCTTCAGCGCAAGAAGAATAG	2988	*fosB*-1th-surround
Between *vanSH*-R	GACTTGGATTGGCGATTGCA		
fosB-2th-F	TGCCGTAAGACTTCCTGAGTTG	3495	*fosB*-2th-surround
fosB-2th-R	CTGAGATACGAAGTTTCCAAAGG		
Q-*fosB*-F	CTCAATCTATCTTCTAAACTTCCTG	156	*fosB*
Q-*fosB*-R	CGATTTTGAAGATTGGTATAACTGG		
Q-*purK*-F	GATATCCAAGATGCGATTGAC	154	*purK*
Q-*purK*-R	CTTCTAAAACACAGGTTCCTTCTC		
