# Supplementary material for: Identification of Novel Conjugative Plasmids with Multiple Copies of fosB that Confer High-Level Fosfomycin Resistance to Vancomycin-Resistant Enterococci
Source: Front Microbiol. 2017 Aug 15;8:1541. doi: 10.3389/fmicb.2017.01541 (PMC5559704; doi:10.3389/fmicb.2017.01541)
Supplement: Supplementary file 3 [file Table_3.pdf]

TABLE S3 Bacterial growth of 19 transconjugants in the presence and absence of a sub-MIC (1/4 MIC) concentration of fosfomycin

Strains no.	Growth rate (/h)		Max OD		Lag time (h)	
	1/4 MIC fosfomycin	Fosfomycin-free	1/4 MIC fosfomycin	Fosfomycin -free	1/4 MIC fosfomycin	Fosfomycin-free
IA14	**0.15** ± 0.01	**0.72** ± 0.01	**0.24** ± 0.01	**1.15** ± 0.01	**3.78** ± 0.29	**2.09** ± 0.02
A157	**0.14** ± 0.01	**0.76** ± 0.00	**0.26** ± 0.02	**1.16** ± 0.01	**4.23** ± 0.13	**2.05** ± 0.01
A92	**0.73** ± 0.03	**0.90** ± 0.04	**0.47** ± 0.02	**1.20** ± 0.01	**3.99** ± 0.06	**1.94** ± 0.09
A13	**0.56** ± 0.06	**0.96** ± 0.03	**0.40** ± 0.03	**1.17** ± 0.01	**14.92** ± 0.38	**2.33** ± 0.02
A67	**0.53** ± 0.00	**0.80** ± 0.00	**0.44** ± 0.00	**1.24** ± 0.01	**3.16** ± 0.01	**2.10** ± 0.01
B42	**0.61** ± 0.02	**0.77** ± 0.02	**0.44** ± 0.02	**1.35** ± 0.01	**2.90** ± 0.07	**2.12** ± 0.04
A9	**0.53** ± 0.01	**0.93** ± 0.03	**0.37** ± 0.00	**1.15** ± 0.00	**5.08** ± 0.04	**2.49** ± 0.03
A120	**0.32** ± 0.02	**0.90** ± 0.04	**0.26** ± 0.01	**1.18** ± 0.01	**5.89** ± 0.36	**2.08** ± 0.08
19081	**0.30** ± 0.02	**0.94** ± 0.04	**0.25** ± 0.00	**1.17** ± 0.01	**5.29** ± 0.12	**2.06** ± 0.04
A158	**0.28** ± 0.01	**0.92** ± 0.04	**0.26** ± 0.00	**1.20** ± 0.01	**5.67** ± 0.09	**2.11** ± 0.02
A96	**0.31** ± 0.01	**0.97** ± 0.02	**0.29** ± 0.01	**1.18** ± 0.01	**5.03** ± 0.24	**2.12** ± 0.03
IB3	**0.32** ± 0.02	**0.90** ± 0.04	**0.29** ± 0.01	**1.20** ± 0.01	**5.47** ± 0.25	**2.16** ± 0.11
A165	**0.29** ± 0.01	**0.94** ± 0.04	**0.30** ± 0.01	**1.20** ± 0.01	**4.84** ± 0.07	**2.10** ± 0.04
A166	**0.30** ± 0.01	**0.91** ± 0.04	**0.29** ± 0.02	**1.21** ± 0.01	**5.13** ± 0.20	**2.28** ± 0.04
A155	**0.28** ± 0.01	**0.91** ± 0.02	**0.29** ± 0.01	**1.21** ± 0.01	**5.01** ± 0.09	**2.11** ± 0.04
1001	**0.36** ± 0.03	**0.91** ± 0.01	**0.29** ± 0.01	**1.19** ± 0.01	**5.16** ± 0.31	**2.12** ± 0.04
IA110	**0.23** ± 0.02	**0.89** ± 0.06	**0.27** ± 0.01	**1.20** ± 0.01	**6.20** ± 0.30	**2.43** ± 0.12
IA28	**0.42** ± 0.03	**0.95** ± 0.01	**0.28** ± 0.02	**1.13** ± 0.01	**12.17** ± 0.32	**2.51** ± 0.03
A3	**0.35** ± 0.02	**1.07**± 0.03	**0.23** ± 0.01	**1.20** ± 0.01	**4.99** ± 0.12	**2.49** ± 0.05
