# Supplementary material for: Identification of Novel Conjugative Plasmids with Multiple Copies of fosB that Confer High-Level Fosfomycin Resistance to Vancomycin-Resistant Enterococci
Source: Front Microbiol. 2017 Aug 15;8:1541. doi: 10.3389/fmicb.2017.01541 (PMC5559704; doi:10.3389/fmicb.2017.01541)
Supplement: Supplementary file 4 [file Image_1.PDF]

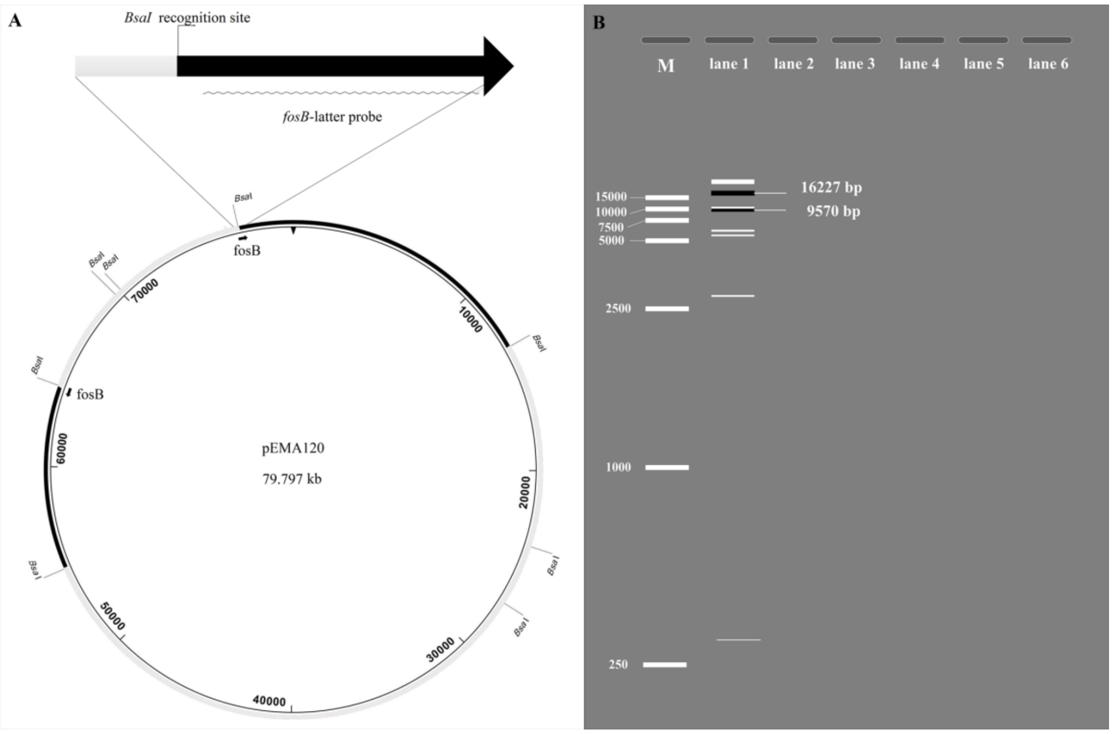

FIGURE S1 | Schematic plot of restriction *BsaI* digestion of pEMA120. (A) *BsaI* recognition sites in plasmid pEMA120. Two fragments carrying the latter part of *fosB* gene are shown in black color. **(B)** Simulating graph of agarose-gel electrophoresis. Eight bands in lane 1 represent eight fragments digested by enzyme *BsaI*. Two fragments, which can combine with *fosB*-latter probe, are colored in black.
