# Supplementary material for: Identification of Novel Conjugative Plasmids with Multiple Copies of fosB that Confer High-Level Fosfomycin Resistance to Vancomycin-Resistant Enterococci
Source: Front Microbiol. 2017 Aug 15;8:1541. doi: 10.3389/fmicb.2017.01541 (PMC5559704; doi:10.3389/fmicb.2017.01541)
Supplement: Supplementary file 5 [file Image_2.PDF]

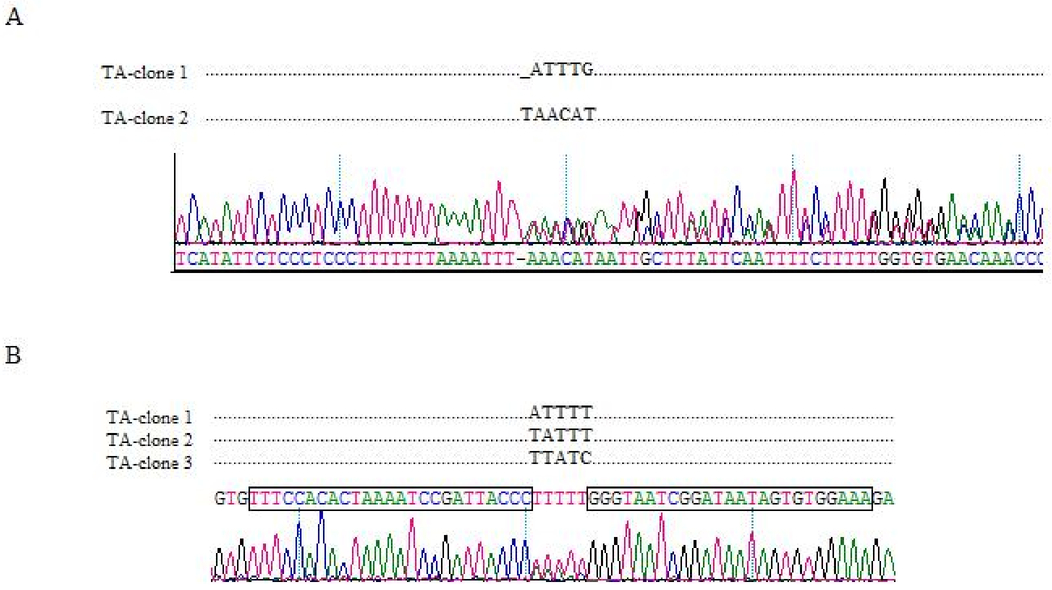

FIGURE S2 | The sequencing electropherograms obtained with primers fosBiF and fosBiR for the PCR product and the pMD20-T TA-clones. (A) The chromatogram of the region 26-31 bp upstream of the fosB gene. **(B)** The chromatogram of the junction region between the two IRs (black boxes).
