# Supplementary material for: Identification of Novel Conjugative Plasmids with Multiple Copies of fosB that Confer High-Level Fosfomycin Resistance to Vancomycin-Resistant Enterococci
Source: Front Microbiol. 2017 Aug 15;8:1541. doi: 10.3389/fmicb.2017.01541 (PMC5559704; doi:10.3389/fmicb.2017.01541)
Supplement: Supplementary file 6 [file Image_3.PDF]

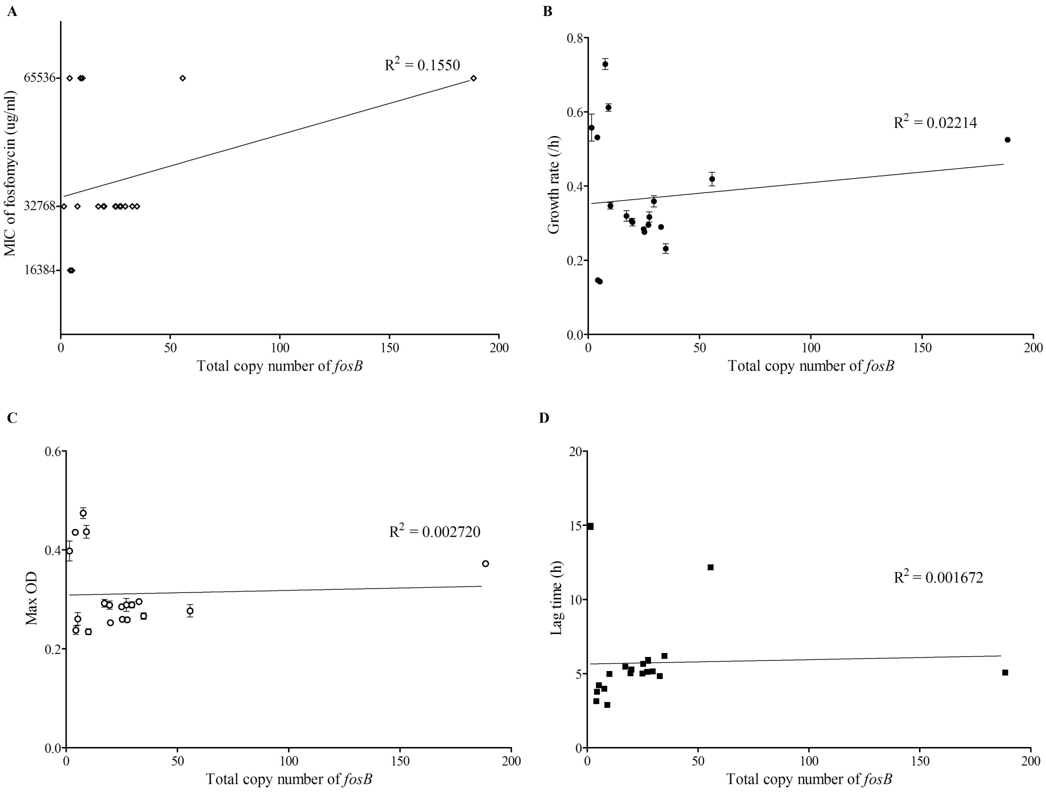

FIGURE S3 | The correlation between copy number of *fosB* and fosfomycin MIC (A), bacterial growth rate (B), max OD (C), and lag time (D) at 1/4 MICs of fosfomycin.
